# PW01-014 – MEFV methylation analysis in FMF and JRA diseases

**DOI:** 10.1186/1546-0096-11-S1-A67

**Published:** 2013-11-08

**Authors:** E Tahir Turanli, AK Kirectepe, Ö Kasapçopur

**Affiliations:** 1 Department of Molecular Biology and Genetics, Istanbul Technical University, Turkey; 2 Molecular Biology-Biotechnology and Genetics Research Center, Istanbul Technical University, Turkey; 3 Department of Pediatric Rheumatology, Cerrahpasa School of Medicine, Istanbul, Turkey

## Introduction

MEFV is the first identified autoinflammatory gene related to Familial Mediterranean Fever (FMF) disease. We previously the tested the hypothesize of epigenetic involvement in FMF, mainly based on the occurrence of FMF in patients without mutations and decreased MEFV transcripts in leukocyte samples independent from mutations. Our study showed that higher methylation level of MEFV second exon CpG island in FMF patients compared to healthy controls (*p*=0.049) and negative correlation between methylation and expression levels in leucocytes (cor=-0.29, *p*=0.041 in both groups, cor=-0.36, *p*=0.035 in FMF samples).

## Objectives

As there are studies suggesting that MEFV might be related not only to FMF, additionally to other inflammatory disorders, we wanted to know our proposed epigenetic involvement hypothesis specificity to FMF. It has also been known that methylation of intronic and exonic sites has a role on regulation of expression by influencing transcription elongation. In this study we aimed to compare CpG island methylation level of MEFV gene in FMF and Juvenile Rheumatoid Arthritis (JRA) patients.

## Methods

DNA was isolated from venous blood of age-gender matched FMF (N=20) and JRA (N=17) patients in attack-free period, who are diagnosed and followed up at Istanbul University, Cerrahpasa Medical Faculty, Department of Pediatric Rheumatology. The parents of children were informed and consent forms were fulfilled. Methylation levels were calculated according to the protocol of OneStep qMethyl Kit (Zymo), which is a real-time PCR procedure based on methylation specific restriction enzyme digestion. The methylation levels were compared between groups using *student-T* test analyses.

## Results

First intron and part of the second exon methylation level of MEFV gene were analyzed in both FMF and JRA groups and there was no significant difference between two groups.

## Conclusion

In this preliminary study we have observed similar MEFV intron 1 and part of exon 2 methylation levels between FMF and JRA patients. In combination with previous studies pointing MEFV involvement in other inflammatory disorders such as JRA and BS, our findings may further support the importance of MEFV in inflammatory pathway and possibly not only through genetic mechanisms but also by means of epigenetic regulations.

## Disclosure of interest

None declared

